# Multi-transcriptomics reveals RLMF axis-mediated signaling molecules associated with bovine feed efficiency

**DOI:** 10.3389/fvets.2023.1090517

**Published:** 2023-03-22

**Authors:** Chaoyun Yang, Yanling Ding, Xingang Dan, Yuangang Shi, Xiaolong Kang

**Affiliations:** Key Laboratory of Ruminant Molecular and Cellular Breeding, School of Agriculture, Ningxia University, Yinchuan, China

**Keywords:** feed efficiency, regulatory axis, consensus analysis, WGCNA, cattle

## Abstract

The regulatory axis plays a vital role in interpreting the information exchange and interactions among mammal organs. In this study on feed efficiency, it was hypothesized that a rumen-liver-muscle-fat (**RLMF**) regulatory axis exists and scrutinized the flow of energy along the **RLMF** axis employing consensus network analysis from a spatial transcriptomic standpoint. Based on enrichment analysis and protein-protein interaction analysis of the consensus network and tissue-specific genes, it was discovered that carbohydrate metabolism, energy metabolism, immune and inflammatory responses were likely to be the biological processes that contribute most to feed efficiency variation on the **RLMF** regulatory axis. In addition, clusters of genes related to the electron respiratory chain, including *ND (2,3,4,4L,5,6), NDUF (A13, A7, S6, B3, B6), COX (1,3), CYTB, UQCR11, ATP (6,8)*, clusters of genes related to fatty acid metabolism including *APO (A1, A2, A4, B, C3), ALB, FG (A, G)*, as well as clusters of the ribosomal-related gene including *RPL (8,18A,18,15,13, P1)*, the *RPS (23,27A,3A,4X)*, and the *PSM (A1-A7, B6, C1, C3, D2-D4, D8 D9, E1)* could be the primary effector genes responsible for feed efficiency variation. The findings demonstrate that high feed efficiency cattle, through the synergistic action of the regulatory axis **RLMF**, may improve the efficiency of biological processes (carbohydrate metabolism, protein ubiquitination, and energy metabolism). Meanwhile, high feed efficiency cattle might enhance the ability to respond to immunity and inflammation, allowing nutrients to be efficiently distributed across these organs associated with digestion and absorption, energy-producing, and energy-storing organs. Elucidating the distribution of nutrients on the **RLMF** regulatory axis could facilitate an understanding of feed efficiency variation and achieve the study on its molecular regulation.

## Introduction

Feed efficiency (**FE**) is one of the significant constraints to livestock development, and its improvement is an essential breeding schedule for the sustainability of the beef cattle industry. However, the distribution of nutrients between organs is unclear. Residual feed intake (**RFI**) has been widely used to evaluate **FE** in dairy cattle (1–3) and beef cattle (4, 5). It was described by Koch in 1963, and its linear regression of dry matter intake (**DMI**) and average daily gain (**ADG**) represents the energy sinks (e.g., the requirement for maintenance, growth, lactation, and egg) of the animal at a certain time and fail to correlate with economic traits such as average daily gain, growth rate (6, 7). RFI belongs to moderate heritability [chicken 0.21–0.50 (8, 9), duck 0.24–0.27 (10–12), pig 0.19–0.63 (13, 14), cattle 0.28–0.40 (15, 16), sheep 0.45 (17)], and is negatively correlated with the **FE**, showing that stable genetic gain could be foreseen from **RFI**-based selection. **RFI** is subject to many factors, such as protein turnover, tissue metabolism, stress, digestibility, heat increment, fermentation, physical activity, body composition, and feeding patterns (18–22). However, the specific mechanisms underlying the biology of **RFI** are still underdeveloped. Plenty of experimenters have endeavored to elucidate **RFI** variation in terms of gene expression from a single tissue [e.g., liver (23), skeletal muscle (24), blood (25), adipose tissue (26), rumen epithelium (27), duodenum (5)]. As each study has focused on only individual tissue, it is highly variable and couldn't acquire a coherent conclusion (28). Although studies included multiple tissues, the results obtained do not exhaustively describe the variation in RFI (29).

The digestion and absorption of nutrients is a coordinating process participated by multi-organ. Nutrients are digested in the gastrointestinal tract and absorbed into the blood, and then transported to multiple organs and tissues such as the liver and muscle to supply energy, with excess nutrients stored as triglycerides in adipose tissues or glycogen in the liver and muscle. Therefore, the process of nutrient intake to digestion, energy supply, and storage must be done collaboratively among tissues, during which different tissues control energy allocation by regulating the expression of functionally related genes and transmitting signals directly or indirectly to other tissues. Indeed, a number of studies have recently shown that the brain-gut axis can modulate obesity and appetite (30) and maintain the homeostasis of gastrointestinal glucose (31). It has also been shown that the liver-brain-gut axis (32), the gut-liver-muscle axis (33), and the muscle-liver-fat axis (34) are closely related to metabolic function. Consensus module analysis based on weighted correlation network analysis (**WGCNA)** is a methodology for identifying and excavating genes associated with complex phenotypic traits and has been used to uncover the metabolites and gene signatures (35, 36). Meanwhile, both consensus module analysis and spatial transcriptomics could be used to explore similarities and differences between tissues. With the limitations of the previous studies, this project is based on the existence of a rumen-liver-muscle-fat (**RLMF**) regulatory axis and performed consensus network construction using multi-transcriptomic data from the rumen, liver, muscle and adipose tissues with divergent RFI to identify consensus and tissue-specific modules, and uncover the eigengenes and biological processes. The objectives of this project are to elucidate the changes in genes and related biological processes from the perspective of nutrient intake and digestion (rumen)-processing/transfer (liver)-energy supply (muscle)-energy storage (adipose). It will provide a well-defined understanding of the contribution of **FE** variation and present the landscape of energy transfer from the gene expression profile.

## Materials and methods

### Data preparation and processing

The GSE116775 dataset was downloaded from the Gene Expression Omnibus (https://www.ncbi.nlm.nih.gov/geo/query/acc.cgi?acc=GSE116775). The present study (29) used four tissues (rumen, liver, fat, and muscle tissue) from the Angus cattle with diverse **RFI** (sequencing platform: Illumina HiSeq 4000; Library layout: pair-end; Library source: TRANSCRIPTOMIC; Average length: 200), including eight animals with high or low **RFI**. After the original file (*fastq* format) was downloaded, the fastqc software (37) (version 0.11.7, https://www.bioinformatics.babraham.ac.uk/projects/fastqc/) and Trim-galore (version 0.6.6, https://www.bioinformatics.babraham.ac.uk/projects/trim_galore/, **parameters**: paired, –quality 25 –length 36, stringency 3) were used to control the quality of reads, respectively. The read fragment with a quality score greater than Q25 was reserved. The quality-controlled clean reads were then aligned (**hisat2**, version 2.2.1, http://daehwankimlab.github.io/hisat2/; **parameters**: default parameters) to the index file of the bovine reference genome ARS-UCD1.2 (downloaded from the *BovineGenome.org* website, https://bovinegenome.elsiklab.missouri.edu/downloads/ARS-UCD1.2) to obtain a *sam* file containing the aligned information. The *sam* files were converted to the *bam* files using samtools software (38) (version 1.9, https://sourceforge.net/projects/samtools/files/samtools/1.9/; **parameters** : -b -S -h) and the *bam* file index was constructed, following gene quantification using the featureCounts program in the subread package (39) (version 2.0.1, http://subread.sourceforge.net/; **parameters**: -t exon -g gene_id ) to acquire the count matrix. Considering that **TPM** (Transcripts Per Kilobase of exon model per Million mapped reads) is an excellent method for quantitating **RNA** abundance and is proportional to the average relative RNA molar concentration, and is used by many computational algorithms for transcript quantification [e.g. RSEM (40) and Salmon (41) methods], we used **TPM** for gene quantification. The formula for **TPM** is: 10^6^^*^ [(reads mapped to transcript /transcript length) /Sum (reads mapped to transcript /transcript length)].

To filter out genes with low and abnormal expression, the Median Absolute Deviation (**MAD**) variable was adopted to remove genes with abnormal TPM values [the advantage of **MAD** for removing abnormal values over the mean and/or standard deviation method (42)] as an input for constructing the network using the **WGCNA** package (43). Initially, the MAD value of all genes was calculated; then all genes in the first quartile (**)** were retained, and finally, all genes with a **MAD** >1 and the overlaps in the two tissues were used to construct the consensus module. Through the **MAD** method, we finally determined that the number of genes that fulfilled the conditions in the rumen, liver, muscle, and fat were 10,868, 8,574, 9,060, and 13,116, respectively. In the consensus gene set, 7,168 (Supplementary Table S1), 6,471 (Supplementary Table S2), 7,621 (Supplementary Table S3), and 8,386 (Supplementary Table S4) genes were used for consensus network construction for rumen-liver (**RL**), liver-muscle (**LM**), liver-fat (**LF**), and muscle-fat (**MF**) regulatory axis, respectively.

In addition to the genes that were used to build the consensus network, tissue-specific genes in the tissue-tissue regulatory axis were selected as tissue-specific genes according to the **MAD** value of top300, and functional enrichment analysis was performed: **RL** (3,700 rumen-specific genes, Supplementary Table S5; 1,406 liver-specific genes, Supplementary Table S6); **LM** (2,103 liver-specific genes, Supplementary Table S7; 2,589 muscle-specific genes, Supplementary Table S8), **LF** (953 liver-specific genes, Supplementary Table S9; 2,028 fat-specific genes, Supplementary Table S10); **MF** (674 muscle-specific genes, 4370 fat-specific genes).

### Network construction and module detection

The **WGCNA** methodology was adopted for the network construction and identification of the consensus module. Before proceeding with the network construction, sample outliers were checked. The Euclidean distance between samples was calculated using the *hclust* function in the **WGCNA** package, with the parameter method = “average”, and samples with distinct outliers were removed. The construction of a weighted gene network requires the optimal selection of soft thresholding power β that improves co-expression similarity and calculates the adjacency. Therefore, picking the optimal soft thresholding power β was performed using the function *pickSoftThreshold* (based on the criterion of approximate scale-free topology) in the R package **WGCNA** (44).

After eliminating the outliers and yielding the optimal soft thresholding power **β**, the function *blockwiseConsensusModules* was employed to calculate the consensus topological overlap and produce the consensus module. Here, *power* = soft thresholding power **β** was used (when R = 0.85). The module contained 30 genes was used as the minimum number (*minModuleSize* = 30), the module detection sensitivity was 2 (*deepSplit* = 2), and the cut height for merging of modules was 0.25 (*mergeCutHeight* = 0.25, i.e., merge into one module if the correlation coefficient of eigengenes within the module is >0.75). In order to avoid rearrangement of eigengene within modules according to **KME**, the parameter *minKMEtoStay* was set to 0, and the parameter *maxBlockSize* was set to 10,000, and the remaining parameters follow the default value of the function.

The acquisition of the tissue-tissue consensus module of interest correlates with the tissue-specific co-expression module of the concern (e.g., Rumen-specific is correlated with the Rumen-Liver consensus module). With this, the hypergeometric test (Fisher's exact test) was used to check the overlap between tissue-specific and tissue-tissue consensus modules (e.g., Rumen-specific and Rumen-Liver), and the correlation between tissue-specific and tissue-tissue consensus modules was derived.

Subsequently, the consensus module was analyzed concerning the **RFI**. First, the co-expression networks for each of the objects used in the consensus network calculation were associated with the **RFI** (e.g., Rumen-specific and Liver-specific co-expression networks in the Rumen-Liver consensus module). The Tissue-specific co-expression network was built using the function *blockwiseModules*, where the parameters are: power = soft thresholding power **β**. *TOMType* = “unsigned”, the *minModuleSize* = 30. *mergeCutHeight* = 0.25. *maxBlockSize* = 20,000. *pamRespectsDendro* = FALSE, verbose = 3, other parameters were set as default. This process yielded tissue-specific co-expression modules associated with **RFI** (significant correlations). When the correlation coefficients used for the two tissue-specific and RFI share the same sign (zero relationships if the two correlations have opposite signs, labeled the “NA”), the minimum correlation coefficient and the maximum significance test *p*-value were preserved to evaluate the relationship between the two modules. The process allows for the unification of the common traits and similarities between the two modules.

Finally, the gene significances (**GS**) and module-memberships (**MM**, also known as **KME**) of eigengenes in the tissue-specific consensus module were calculated using the function *corAndPvalue*. For the purpose of measuring the relationship among all genes in the two tissue-specific co-expression modules, a “meta-analysis” was carried out to establish their correlations. Once the consensus network modules were obtained, the genes within the significantly related modules of the consensus or tissue-specific network were subjected to functional enrichment analysis to elucidate the biological processes and signaling pathways, and protein-protein interaction (***PPI***) analysis was conducted to discover the core genes and key regulatory sub-networks (If there are multiple non-significantly correlated modules, the top 3 are selected according to the absolute value of the correlation coefficient; also if the number of genes in the module was more significant than 300, the top 300 absolute values of **GS** are selected for subsequent analysis).

### PPI and key gene analysis

Protein-protein interactions (**PPI**) were obtained using the *Strings* website (https://string-db.org/, version 11.0) with the following parameters; Organism: *Bos taurus;* minimum required interaction score was set to high confidence (0.7), other parameters were set to default. The CytoHubba plugin in Cytoscape was used to detect hub genes through four centrality methods which were network topology analysis—**Degree**, edge percolated component (**EPC**), maximal clique centrality (**MCC**), and maximum neighborhood component (**MNC**), which are practical methods for identifying hub gene from PPI networks (45). The overlaps of the four methods (the highest top20) were defined as hub genes. The **MCODE** plugin in Cytoscape was applied to identify critical sub-networks and the seeds of nodes (the seeds of nodes were also defined as hub genes), and the parameter configuration is ***degree cutoff*** = *2*, ***node score*** ***cutoff*** = *0.2*, ***k-core*** = *2*, and ***maximum depth*** = *100*. Subsequently, genes from key sub-networks were subjected to functional enrichment analysis.

### Gene function classification and annotation

The vast majority of present functional annotation programs are updated slowly, leading to most of the results are missed (e.g., the most popular David website has data annotation information prior to 2016, which does not explain the results of the latest studies properly) (46). Therefore, the R package clusterProfiler (version 4.05) was designed. The clusterProfiler package is dependent on the genome-wide annotation packages (OrgDb) project published by Bioconductor, which is updated semi-annually, i.e., the gene functions that we could annotate here are the latest and most up-to-date versions available (47). The enrichGO function was applied to the annotation of Gene Ontology, which includes a biological process (**BP**), molecular function (**MF**), and cellular component (**CC**), where the parameters are set as follows: *pvalueCutoff* = 0.05 (adjusted *P*-value cutoff on enrichment tests), *qvalueCutoff* = 0.2 (q-value cutoff on enrichment tests), *pAdjustMethod* = “BH” (multiple test correction method for *p*-values, i.e., Benjamini & Hochberg method), and the maximum number of genes enriched in the pathway *maxGSSize* and the minimum number *minGSSize* are adjusted according to the size of the annotated gene set. The enrichKEGG function was adapted to the Kyoto Encyclopedia of Genes and Genomes (**KEGG**) annotation to uncover the relevant signaling pathways with the same parameters as the enrichGO function. All enrichment analysis results were visualized using the R package ggplot2.

## Results

### Consensus module detection

**Figure 1:**
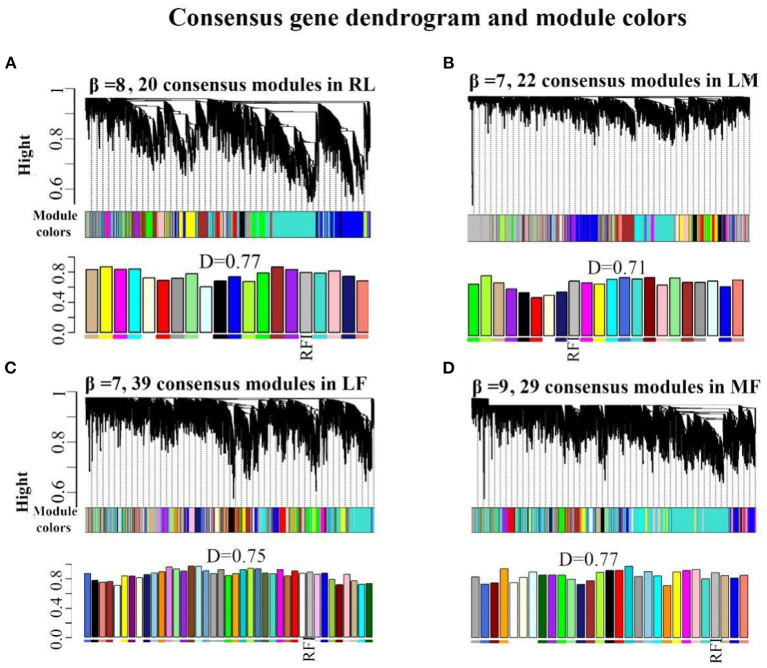
Consensus module construction and detection. **(A–D)** was the consensus module construction for RL, LM, LF, and MF, and its power β, module amount and preservation were (8,7,7,9), (20,22,39,29) and (0.77, 0.71, 0.75, 0.77), respectively.

When the scale-free topology model fit reached 0.85 (R=0.85), a soft thresholding power (β = 8,7,7,9) was assigned to construct the rumen-liver (**RL**), liver-muscle (**LM**), liver-fat (**LF**), and muscle-fat (**MF**) consensus module (Supplementary Figures S1A–D). As a result, several consensus modules (20,22,39, and 29 modules in RL, LM, LF, MF regulatory axis, respectively) were detected, and its total preservation of the two eigengene networks was more than 0.71, indicating that there was considerable similarity between the eigengene co-expression modules (Figures 1A–D).

For RL regulatory axis (Supplementary Figure S2A and Table 1), three consensus modules (turquoise, midnightblue, tan), three rumen-specific modules (green, black, red), and a liver-specific module (lightcycan) were detected. For LM regulatory axis (Supplementary Figure S2B and Table 1), three consensus modules (purple, yellow, pink), three muscle-specific modules (midnightblue, salmon, red), and a liver-specific module (lightyellow) were observed as significant correlation modules. For LF regulatory axis (Supplementary Figure S3A and Table 1), two significant correlation consensus modules (sienna3, white), a liver-specific module (grey60), and three fat-specific modules (blue, lightcyan, plum1) were detected. For MF regulatory axis (Supplementary Figure S3B and Table 1), three significant correlation consensus modules (turquoise, yellow, darkgreen), three muscle-specific modules (brown, orange, darkorange); and three fat-specific modules (royalblue, lightcyan, tan) were identified. These results indicated that in the regulatory axis RLMF, in addition to the modules with similar expression patterns, there were also tissue-specific modules, suggesting that the four tissues may have functional similarities and specificity.

**Table 1:** Names of detected modules and its correlation coefficient in the RLMF regulation axis.

**Axis name**	**Module source**	**Module**	**Correlation**	***P**-value*
			**Coefficient**	
RL axis	Consensus	Turquoise	0.48	0.06
	Consensus	Midnightblue	0.48	0.06
	Consensus	Tan	−0.47	0.07
	Rumen-specific	Green	0.63	0.01
	Rumen-specific	Black	0.74	0.002
	Rumen-specific	Red	0.58	0.03
	Liver-specific	Lightcycan	−0.47	0.06
LM axis	Consensus	Purple	0.53	0.03
	Consensus	Yellow	0.46	0.07
	Consensus	Pink	−0.48	0.06
	Muscle-specific	Midnightblue	0.71	0.001
	Muscle-specific	Salmon	0.76	0.001
	Muscle-specific	Red	0.7	0.003
	Liver-specific	Lightye-llow	−0.67	0.03
LF axis	Consensus	Sienna3	0.55	0.03
	Consensus	White	0.66	0.006
	Liver-specific	Grey60	−0.63	0.001
	Fat-specific	Blue	0.58	0.04
	Fat-specific	Lightcyan	0.69	0.007
	Fat-specific	Plum1	0.61	0.02
MF axis	Consensus	Turquoise	0.65	0.003
	Consensus	Yellow	0.63	0.02
	Consensus	Darkgreen	−0.63	0.01
	Muscle-specific	Orange	−0.79	0.002
	Muscle-specific	Brown	0.74	0.001
	Muscle-specific	Darkorange	0.76	0.001
	Fat-specific	Royalblue	0.56	0.04
	Fat-specific	Lightcyan	0.56	0.05
	Fat-specific	Tan	0.6	0.03

### Enrichment and PPI analysis of the RL regulatory axis and rumen- or liver-specific module

#### Enrichment analysis for Consensus module in RL regulatory axis

**Figure 2:**
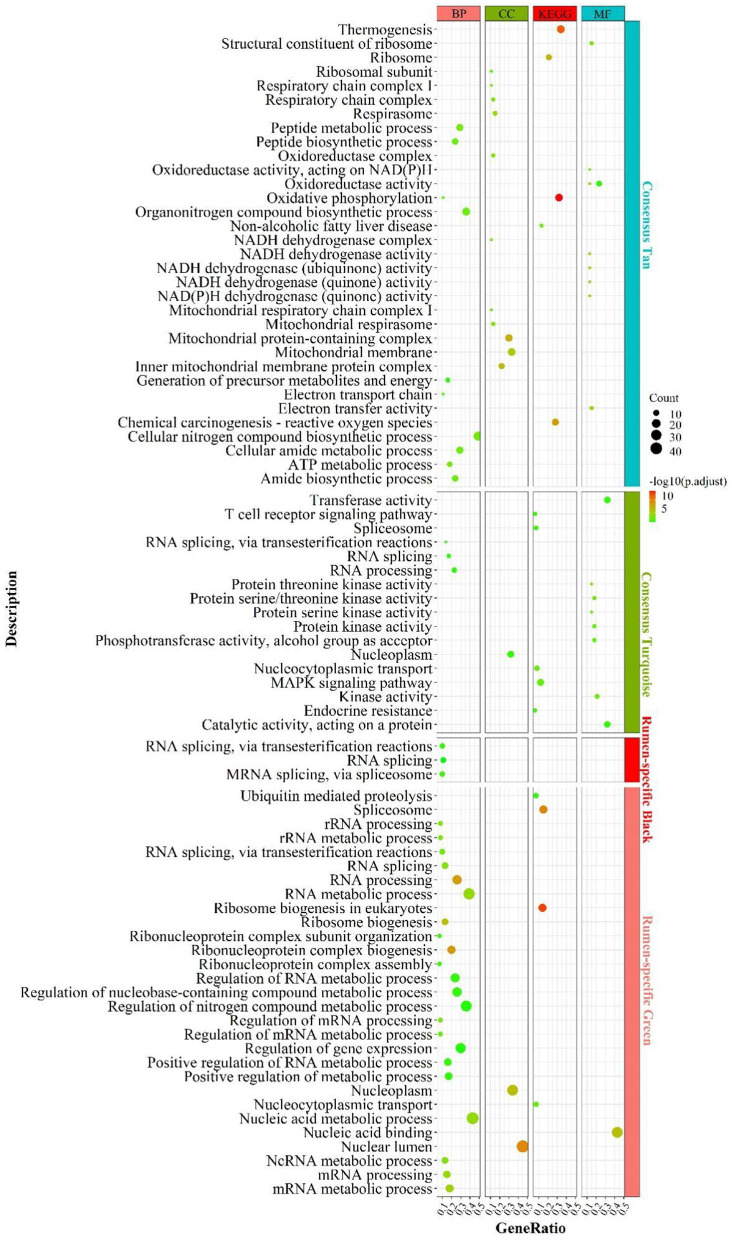
Enrichment analysis for consensus or rumen-/liver-specific modules in **RL** regulatory axis. The dot size and color represented enriched gene numbers and the enriched significance.

Functional enrichment analysis of the genes contained in the consensus eigengene networks was carried out to unravel the biological functions of eigengenes (Figure 2 and Supplementary Table S11). The turquoise consensus module is primarily involved in executing molecular functions such as catalytic activity, kinase activity, transferase activity, etc. It is also involved in disease and inflammation-related signaling pathways such as “MAPK signaling pathway”, “T cell receptor signaling pathway”, etc. Biological processes are mainly associated with RNA molecular pathways, such as “spliceosome”, “RNA splicing”. In the tan consensus module, the genes were mainly involved in pathways related to energy metabolism, such as “oxidative phosphorylation”, “chemical carcinogenesis—reactive oxygen species”, “thermogenesis”; biological processes such as “ATP metabolic process”, “electron transport chain”, “respiratory electron transport chain”, “ATP synthesis coupled electron transport”, etc.; molecular functions were primarily performed by respiratory chain-related enzymes such as “NADH dehydrogenase activity”, “NADH dehydrogenase activity”, “oxidoreductase activity”, “acting on NAD(P)H, electron transfer activity”, etc.; the cellular components are mainly located in mitochondrial-related structures such as the “mitochondrial membrane”, the “mitochondrial proton-transporting ATP synthase complex”, and the “mitochondrial respiratory”. The other consensus module, midnight-blue consensus, was not enriched for the relevant biological pathways or processes under the set threshold conditions.

The results of eigengenes' enrichment analysis for the consensus module above suggested that three main functionally similar findings in RL regulatory axis. The first was that energy metabolism was widespread. The second was that the eigengenes in the consensus module activated the corresponding signaling pathways in response to inflammation or pathology. The third was that given the diverse function of rumen and liver, RNA function-related pathways were activated to adapt to the complex environment.

#### Enrichment analysis for rumen- or liver-specific modules

Meanwhile, rumen-specific module enrichment analysis showed that green and black modules were mainly involved in ribonucleotide-related biological processes, such as nucleic acid metabolic process, RNA metabolic process, ribosome biogenesis, regulation of nitrogen compound metabolic process, positive regulation of metabolic process; and ubiquitin-mediated proteolysis and binding, ubiquitin-dependent protein binding (Figure 2 and Supplementary Table S11).

In addition to the genes used in the non-consensus module, the genes with a **MAD** value of the top 300 in the rumen are primarily enriched in the regulation of epithelial cell motility and movement (Supplementary Figure S4), such as “cell motility” and “epithelium development”. The genes with a MAD value of top 300 in the liver were predominantly enriched in inflammation-related signaling pathways such as “inflammatory response”, as well as in fatty acid degradation pathways (“PPAR signaling pathway”, “fat digestion and absorption”) and glucose and amino acid metabolism. These suggest that the rumen-specific genes were mainly involved in mRNA-related processes and related pathways such as cell motility, which may be required for rumen motility.

#### Protein-protein interaction and hub gene analysis

**Figure 3:**
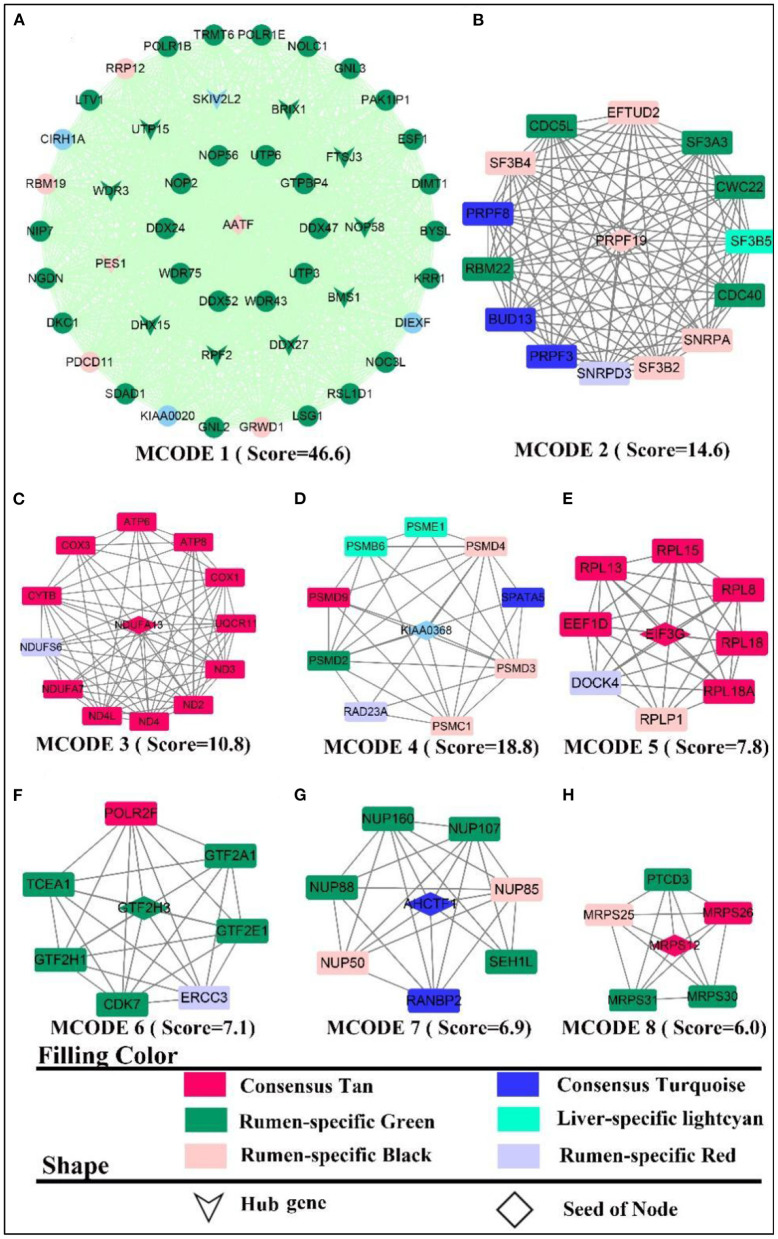
PPI and hub gene analysis for genes in **RL** regulatory axis. The color and shape represented gene source and gene type, respectively.

By elucidating the interactions between genes, it revealed the interactions network contained 1,243 nodes, 3,990 seeds, and eight sub-networks with a score value >6 (Figures 3A–H). Eleven hub genes were also discovered (Figure 3A, all from rumen-specific module green), which were *DDX27, BRIX1, FTSJ3, BMS1, UTP15, RPF2, PES1, WDR3, NOP58, SKIV2L2*, and *DHX15*. The eight subnetworks in which the genes were at the hub were *AATF, PRPF19, NDUFA13, KIAA0638, EIF3G, GTF2H3, AHCTF1, MRPS12*. The enrichment analysis revealed that the genes, in the consensus module tan, primarily involved in energy metabolism-related pathways. After PPI analysis, we detected a cohort of genes associated with the cellular electron transport chain (Figure 3C), including NADH-related genes *ND (2,3,4), NDUFA (7,13, S6)*, cytochrome-related genes *COX (1,3), CYTB, UQCR11)*, and ATP synthase-related genes *ATP (6,8)*. Meanwhile, a set of ribosome-function related genes, *RPL (1,8,18A,18,15,13*), constituted another sub-network (Figure 3E). The rumen-specific green module was predominantly engaged in RNA biological processes, which were consistent with the enrichment analysis results. These genes consisted of DEAD-box decapping family genes (Figure 3A), *DDX* (*24,27,47,52*), *DHX15*; ribosomal function-related genes (Figure 3A), *UTP* (*3,6,15*), *WDR* (*3,43,75*); transcription-related genes (Figure 3F), *GTF2(A1, E1, H1, H3), POLR2F, TCEA1, ERCC3*; protein degradation related genes (Figure 3D), *PSM* (*E1, D2-4, D9, C1, B6*); nuclear pore complex protein gene *NUP* (*50,85,88, 160,107*). PPI analysis of liver-specific top 300 and rumen-specific top 300 genes were identified by MCODE with Score = 9.3 (Supplementary Figure S5), in which the genes were all from liver-specific top 300 and a cohort of genes associated with fatty acid transport, *APO (A1, A2, B, C3, H), ALB, AMBP, FABP1*.

In summary, the integration of enrichment and PPI analyses demonstrated that respiratory-related genes and pathways become active in both the liver and the rumen, which explains that the two tissues participate in energy-intensive activities such as peristalsis, nutrient uptake and transport, as well as the transport of substances and the breakdown of some macro-molecules. In addition, as there was various heterotrophic microbe colonized in the rumen, they provided small molecule nutrients for themselves and the ruminants by breaking down the ingested large molecules, and this process also required energy from respiration. Meanwhile, signaling pathways related to energy metabolism were also more active in the liver, which might be due to its distinctive energy-center and detoxification-center properties.

### Enrichment and PPI analysis of the LM regulatory axis and liver- or muscle-specific module

#### Enrichment analysis for Consensus module in LM regulatory axis

**Figure 4:**
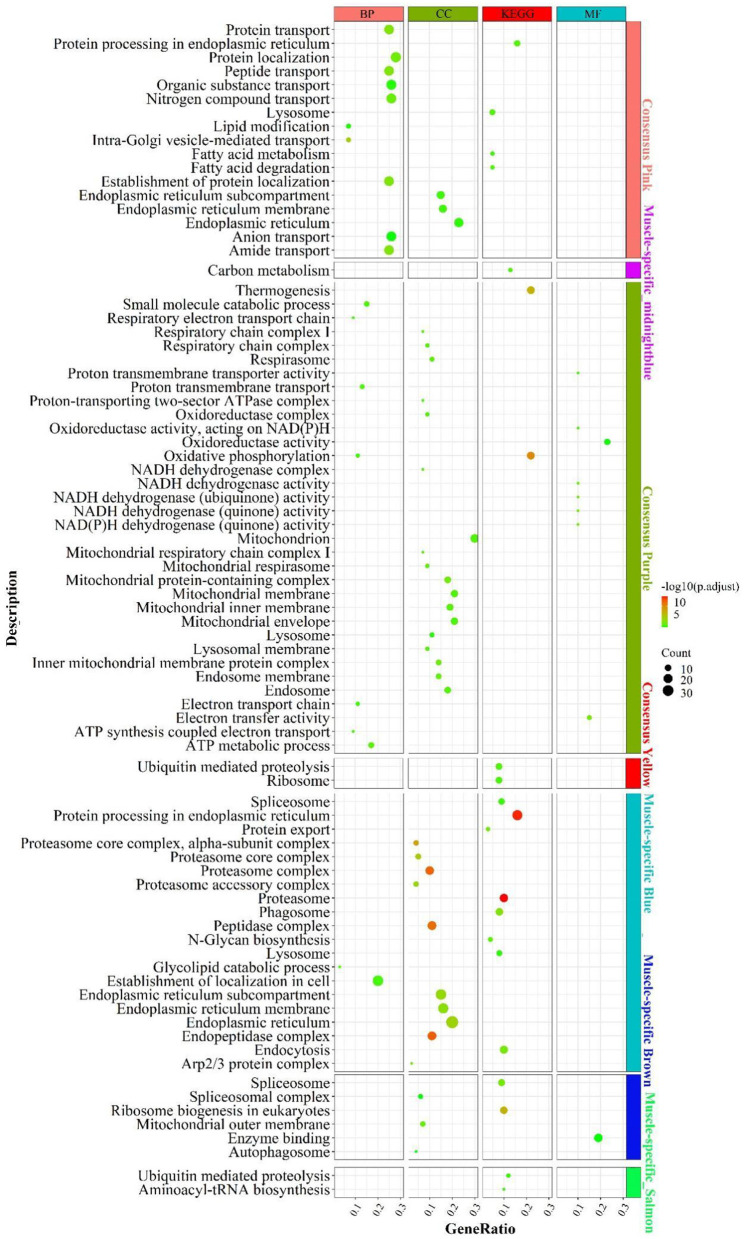
Enrichment analysis for consensus or liver-/muscle-specific modules in **LM** regulatory axis. The dot size and color represented enriched gene numbers and the enriched significance.

According to the enrichment analysis (Figure 4), the genes of the consensus-pink module were principally enriched in pathways involved in lipid catabolism, such as “fatty acid degradation”, “fatty acid metabolism”, “lipid modification”; Protein function-related biological processes, such as “amide/peptide/protein transport”, “protein processing in the endoplasmic reticulum”, “negative regulation of protein tyrosine kinase activity”. In the consensus-purple module, genes were primarily enriched in pathways linked to energy metabolism, such as mitochondrial-related function “mitochondrial respiratory”, “mitochondrial respiratory chain complex I”, “mitochondrial proton-transporting ATP synthase complex”; electronic transmission , such as “electron transport chain”, “energy coupled proton transport, down electrochemical gradient”, “respiratory electron transport chain”; oxidative phosphorylation, such as “oxidative phosphorylation”, “oxidoreductase complex”, “oxidoreductase activity”; cellular respiration, such as “mitochondrial respiratory”, “respiratory chain complex I”, “respiratory electron transport chain”, NADH activity, such as “NADH dehydrogenase activity”, “NADH dehydrogenase (quinone) activity”, “NADH dehydrogenase complex” and ATP and calorie formation, such as “ATP biosynthetic process”, “ATP synthesis coupled electron transport”, “ATP metabolic process”, “thermogenesis”. The consensus module yellow was mainly enriched in “ribosome” and “ubiquitin mediated proteolysis”.

#### Enrichment analysis for liver- or muscle-specific modules

In the muscle-specific module midnightblue (Figure 4), the genes were chiefly enriched in “carbon metabolism”. In the muscle-specific module blue, genes were primarily involved in pathways related to protein metabolism, such as “protein export”, “protein processing in endoplasmic reticulum”; proteasome complex phagocytosis, such as “endocytosis”, “phagosome” and “lysosome”. Genes in muscle-specific module brown mainly enriched in RNA processing, such as “spliceosome complex”, “spliceosome”, ribosome biosynthesis “ribosome biogenesis in eukaryotes; “autophagy-related “autophagosome”. The dominant enrichment in the muscle-specific module salmon was in “aminoacyl-tRNA biosynthesis” and “ubiquitin mediated proteolysis”.

In addition to the genes used in the non-consensus module (Supplementary Figure S6), the genes with a MAD value of top 300 in the liver were primarily enriched in inflammation-related signaling pathways (“e.g., inflammatory response”, “adaptive immune response”), protein-related (e.g., “proteolysis”, “enzyme regulator activity”), carbohydrate metabolism-related (e.g., “PPAR signaling pathway”, “carbohydrate metabolic process”, “pentose and glucuronate interconversions”). In muscle, the genes with a MAD value of top 300 are mainly enriched in muscle contraction (e.g. “calcium signaling pathway,” “muscle contraction”) and muscle development-related pathways (e.g. “skeletal muscle organ development,” “muscle cell differentiation”). Accordingly, the main differences between liver and muscle was mainly the more active catabolism of the three major nutrients and the inflammatory response-related functions in the liver, whereas muscle-specific genes were mainly associated with the development of muscle contraction, as well as the way substances enter cells: cytokinesis and cytokinesis, and the activation of cellular autophagy signaling pathways caused by the possible production of ROS after the muscle has undergone a large amount of oxidative energy supply.

#### Protein-protein interaction and hub gene analysis

**Figure 5:**
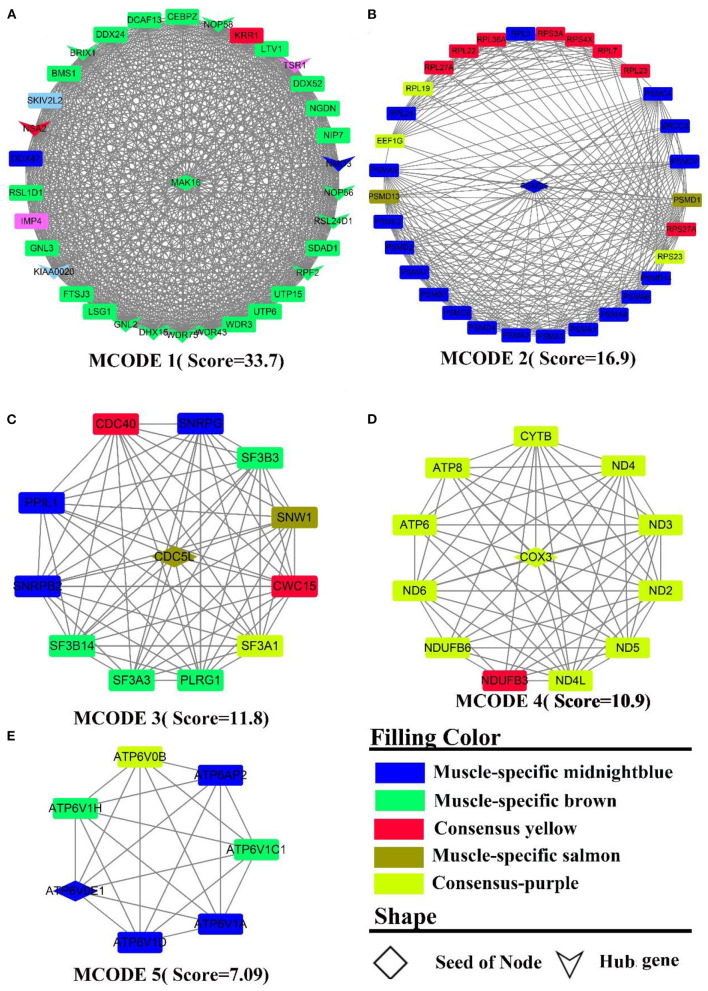
PPI and hub gene analysis for genes in **LM** regulatory axis. The color and shape represented gene source and gene type, respectively.

PPI analysis was adopted to explicate a cluster of structurally and functionally similar genes. By analyzing the interactions between genes, 931 nodes, 3,863 edges were derived. Five sub-networks with a score >6 (Figure 5) and 14 hub genes (the shape “V” in Figure 5A) were also detected, namely *BRIX1, DDX24, DHX15, GNL2, KIAA0020, NMD3, NOP56, NOP58, NSA2, RPF2, RSL24D1, TSR1, WDR43, WDR75*. The genes in MCODE 4 (Figure 5D) were all derived from the consensus module purple, and PPI analysis identified clusters of genes related to energy metabolism, such as *ND(2-6,4L), ATP (6,8), COX3, CYTB*, and *NDUFB (3,6)*. MCODE 2 (Figure 5B) was composed primarily of muscle-specific module midnightblue and consensus module yellow genes, which were mainly involved in protein turnover (e.g., protein ubiquitination). These gene clusters include *PSMA(1-7)*, the proteasome-related gene clusters *PSM (C4, C6, D1-2, D4-7, D13-14)*, and the ribosomal function-related gene clusters *RPL(3,7,19,22-24,27A,36A)* and *RPS (23, 27A, 3A, 4X)*. In MCODE 1 (Figure 5A, muscle-specific module brown), the genes were mainly involved in ribosome-related functions, such as *UTP(6,15), WDR(3, 43, 75), RSL(1D1, 24D1), NOP(56, 58), BMS1, BRIX1*, and transcription-related genes, such as *DCAF13, DDX(24,52), DHX15, CEBPZ, NIP7*, which was identical to the results of the enrichment analysis. In MCODE 3(Figure 5C), genes were mainly involved in mRNA processing, such as *SF3(A1, A3, B3, B14), CWC15*. In MCODE 5 (Figure 5E), genes were primarily derived from muscle-specific midnightblue modules, and principally involved in energy metabolism, e.g., *ATP6(V1H, AP2, V1A, V0B, V1D, V0E1, V1C1)*. PPI analysis of liver-specific top 300 and muscle-specific top 300 genes revealed three MCODEs with Scores >6 (Supplementary Figure S7), which contained genes all from the liver. MCODE 1 (Supplementary Figure S7A) contained the lipoprotein-related genes *APO (A1, B, C3, H), AHSG, ALB*. MCODE 2 (Supplementary Figure S7B) also contained the lipoprotein-related genes *APO (A2, A4), FABP1, FG (A, G), HP, HPX, LPL, AMBP*. MCODE 3 (Supplementary Figure S7C) contained the UDP-glucuronosyltransferase *UGT (2B15, 2B17, 2B4, 2B10, 1A1)*, and cytochrome-related gene clusters *CYP (4A11, 3A24, 2E1, 1A2)*.

To summarize, as a result of both enrichment and PPI analysis, energy metabolism and protein metabolism were the active biological processes in the **LM** regulatory axis. However, there are distinctions, e.g., more genes in the liver are enriched in inflammation-related pathways, whereas in muscle, they are more involved in muscle cell development and movement. The analysis of the consensus module pointed to the fact that energy metabolism was an essentially biological process for the LM regulatory axis, as both of the organs require considerable energy. For muscle, the contractile movement is a powerful energy-consuming and thermogenic process with the fastest protein turnover.

### Enrichment and PPI analysis of the LF regulatory axis and liver- or fat-specific module

#### Enrichment analysis for Consensus module in LF regulatory axis

There was no result for enrichment during the consensus network analysis of LF regulatory axis.

#### Enrichment analysis for liver- or muscle-specific modules

**Figure 6:**
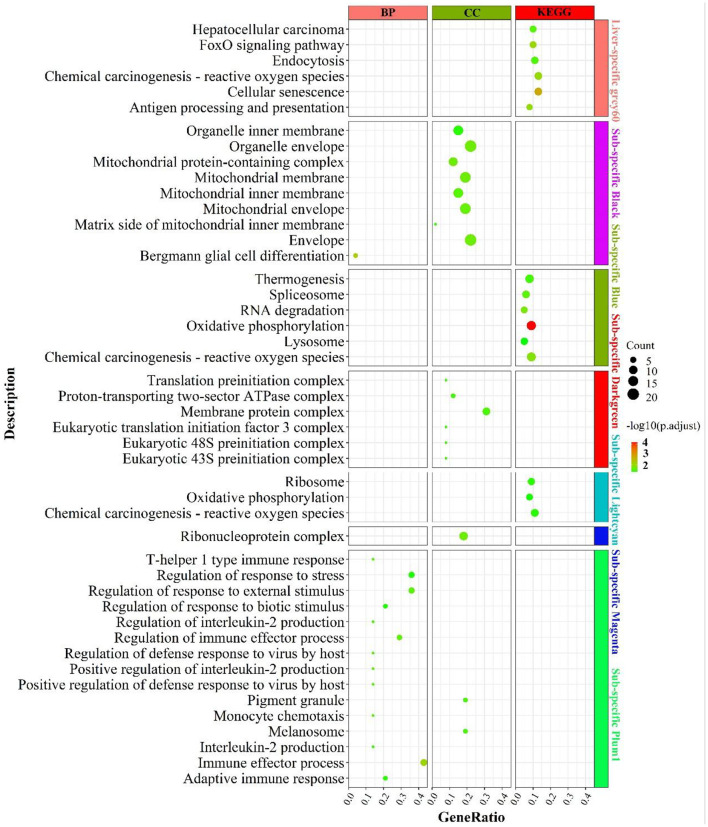
Enrichment analysis for consensus or liver-/fat-specific modules in **LF** regulatory axis. The dot size and color represented enriched gene numbers and the enriched significance.

The enrichment results were shown in Figure 6. Liver-specific grey60 was primarily enriched in cell cycle-related pathways such as “FoxO signaling pathway”, “chemical carcinogenesis—reactive oxygen species”, “cellular senescence” and “endocytosis”. In the fat-specific black module, genes were principally enriched in mitochondria-related cellular components such as “mitochondrial membrane”, “mitochondrial protein-containing complex”, “mitochondrial membrane”, etc. In the fat-specific blue module, genes were abundant in energy metabolism-related pathways, such as “oxidative phosphorylation”, “thermogenesis”, “chemical carcinogenesis - reactive oxygen species” and gene transcription-related pathways such as “spliceosome”, “RNA degradation”. In fat-specific lightcyan, the main enrichment was in “oxidative phosphorylation”, “chemical carcinogenesis - reactive oxygen species”, and “reactive oxygen species”. In fat-specific plum1, genes were primarily involved in inflammation-related biological processes, such as “regulation of immune effector process”, “adaptive immune response”, “interleukin-2 production”, “regulation of interleukin-2 production”.

In addition to the genes used in the non-consensus module (Supplementary Figure S8), genes with top 300 MAD values in the liver were mainly enriched in inflammation-related pathways (“inflammatory response”, “leukocyte mediated immunity”), fatty acid metabolism-related pathways (“PPAR signaling pathway”, “linoleic acid metabolism”, “organic acid catabolic process”), proteolytic metabolism (“Proteolysis”, “negative regulation of proteolysis”). The genes with a MAD value of top 300 in fat were also mainly enriched in the “PPAR signaling pathway”, “fat cell differentiation”, and “aldosterone” and “aldosterone synthesis and secretion”. To summarize, liver-specific genes are mainly involved in biological processes such as energy metabolism, inflammatory response and cell cycle, and longevity; fat-specific genes are mainly involved in energy metabolism and inflammatory metabolism, indicating that the two organs have many operational functions similarities they express different genes.

#### Protein-protein interaction and hub gene analysis

**Figure 7:**
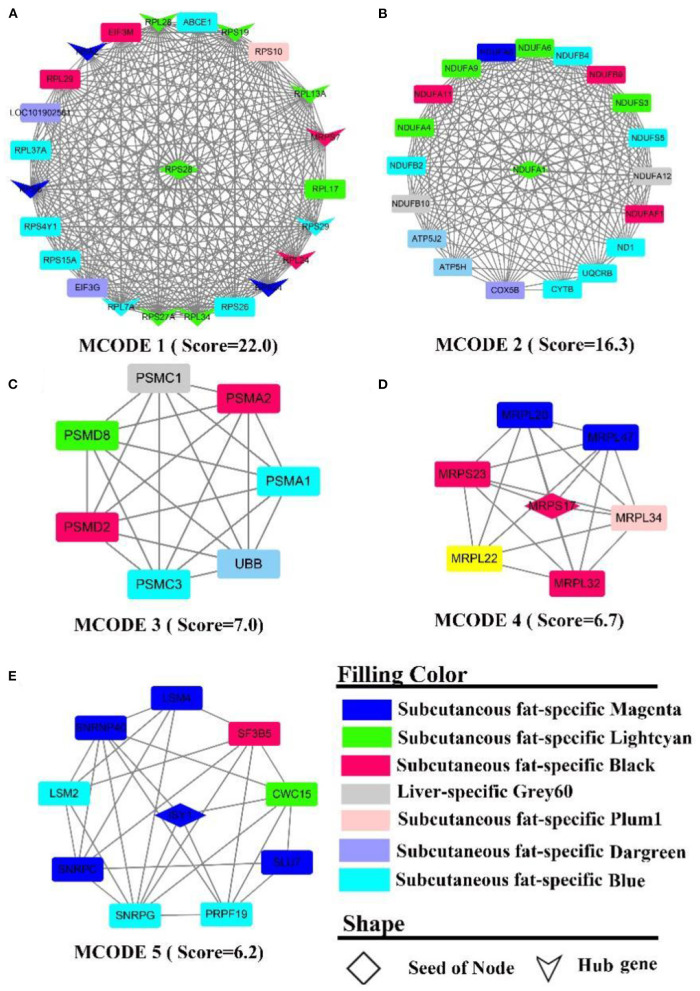
PPI and hub gene analysis for genes in **LF** regulatory axis. The color and shape represented gene source and gene type, respectively.

The PPI network contains 566 nodes, 1,872 seeds, and contains 12 hub genes (Figure 7A, the shape “v”), namely *RPL (7A, 13A, 24, 28, 34, 37A), RPS (24, 19, 27A, 8, 28, 29)*. Five sub-networks were detected in this network. In MCODE 1 (Figure 7A), there are 12 hub genes, and ribosomal function-related gene clusters, *RPS (4Y1, 26, 15A, 10), RPL (29, 17)*. In MCODE 2 (Figure 7B), a similar result to the enrichment analysis identified a cluster of genes that are tightly linked to energy metabolism, such as ubiquinone oxidoreductase-related genes, *ND1, NDUF (A1, A4-6, 9, A11-12, F1, B2, B4, B9, B10, S3, S5), ATP5(H, J2)*, and cytochrome, such as *UQCRB, COX5B, CYTB*. In MCODE 3 (Figure 7C), genes were primarily involved in the ubiquitination of proteins and proteasome, such as *PSM(A1-2, C1, C3, D2, D8), UBB*. In MCODE 4 (Figure 7D), genes were mainly involved in the function of mitochondrial ribosomal proteins, such as genes *MRP (S17, L20, L22, S23, L34, L32, L47)*. In MCODE 5 (Figure 7E), genes were largely involved in the transcriptional processes of genes such as *SF3B5, PRPF19, LSM (2,4)*. MCODE 1 (Score = 8.6) and MCODE 2 (Score = 6.5) were identified by PPI analysis of liver-specific top 300 and fat-specific top 300 (Supplementary Figure S9). MCODE 1 (Supplementary Figure S9A) comprises several genes associated with the adipose metabolism: *ALB, APO (C3, H), FGB, GC, HRG, PAH*. MCODE 2 (Supplementary Figure S9B) also contains multiple genes associated with adipose metabolism, such as *APO (B, A4), FABP1*, and *LPL*.

In summary, although enrichment analysis revealed that the consensus module of LF regulatory axis was not enriched for the relevant biological processes, it indicated that the similarities between liver and fat were low from a gene expression perspective, which may explain the fact that the two tissues perform different functions. However, there were significant similarities between the two tissues external to the consensus network, such as involvement in energy metabolism and inflammation-related pathways.

### Enrichment and PPI analysis of the MF regulatory axis and muscle- or fat-specific module

#### Enrichment analysis for Consensus module in MF regulatory axis

**Figure 8:**
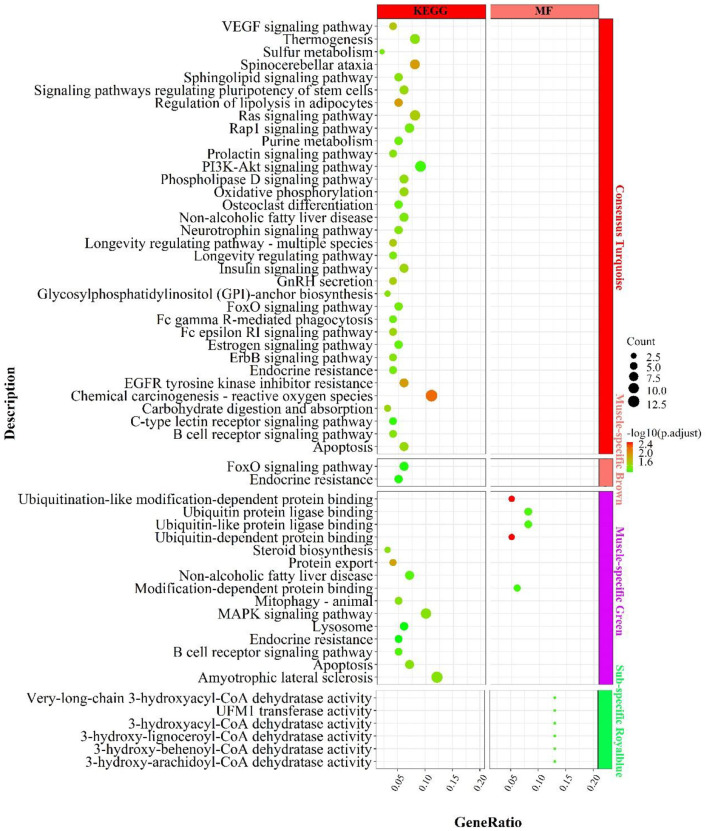
Enrichment analysis for consensus or muscle-/fat-specific modules in **MF** regulatory axis. The dot size and color represented enriched gene numbers and the enriched significance.

In the consensus module turquoise, genes were enriched in signaling pathways related to carbohydrate metabolism (Figure 8), such as “carbohydrate digestion and absorption”„ “regulation of lipolysis in adipocytes”, “phospholipase D signaling pathway”; energy metabolism, such as “oxidative phosphorylation”, “chemical carcinogenesis-reactive oxygen species”; “thermogenesis; inflammation, such as “PI3K-Akt signaling pathway, “ “B cell receptor signaling pathway”, “FoxO signaling pathway”, “insulin-related signaling pathway”, “GnRH secretion”, “neurotrophin signaling pathway”„ “neurotrophin signaling pathway”, etc.

#### Enrichment analysis for liver- or muscle-specific modules

In the muscle-specific module brown (Figure 8), the two major enrichment pathways were “endocrine resistance” and “FoxO signaling pathway”. In the muscle-specific module green (Figure 8), genes were primarily involved in protein ubiquitination-related functions, such as “ubiquitin-dependent protein binding”, “ubiquitin protein ligase binding”, “protein export”, the inflammation-related signaling pathways, such as “MAPK signaling pathway”, “B cell receptor signaling pathway”, “apoptosis”, and the lipid synthesis-related signaling pathway, such as “steroid biosynthesis”.

In the fat-specific module royalblue, genes mainly performed CoA dehydratase activity but had fewer enriched pathways, such as “very-long-chain 3-hydroxyacyl-CoA dehydratase activity”, “3-hydroxyacyl-CoA dehydratase activity”, “3-hydroxy-behenoyl- CoA dehydratase activity”, “3-hydroxy-behenoyl-CoA dehydratase activity”. In addition to the genes used in the consensus module (Supplementary Figure S10), genes with MAD values top 300 in muscle were mainly enriched in the processes of calcium ion involvement in cell contraction , such as “calcium signaling pathway”, “regulation of calcium ion transmembrane transport”, “cAMP signaling pathway”, “regulation of calcium ion transmembrane transport”, “cAMP signaling pathway”, etc.; and skeletal muscle cell contraction, such as “skeletal muscle contraction”, “regulation of muscle system process, “ and signaling pathways that regulate blood glucose concentration, such as “glucagon signaling pathway, “ “insulin secretion”, “cAMP signaling pathway.” In contrast, genes with top 300 MAD values in fat were not enriched to the respective pathways.

#### Protein-protein interaction and hub gene analysis

**Figure 9:**
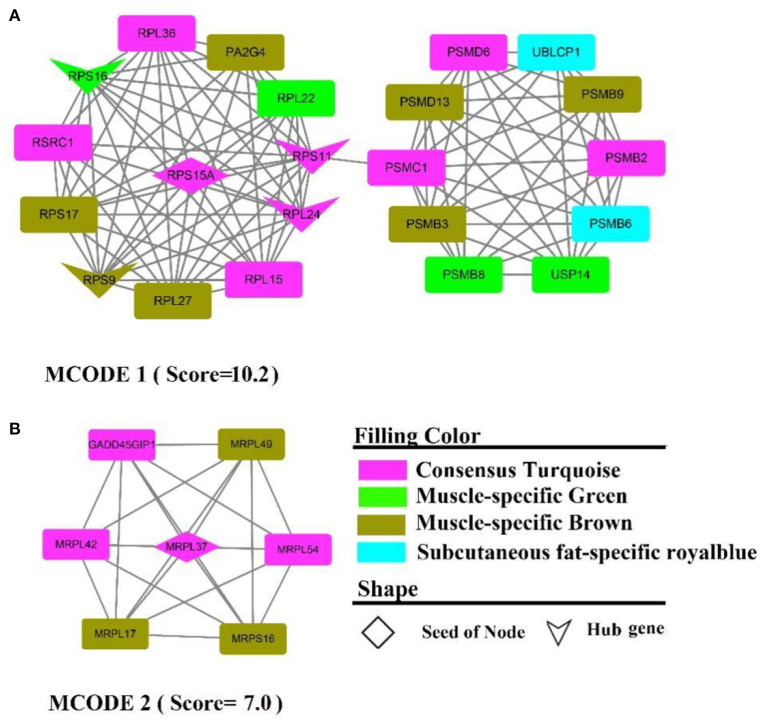
PPI and hub gene analysis for genes in **MF** regulatory axis. The color and shape represented gene source and gene type, respectively.

The PPI network contains 313 nodes and 828 edges, while five core genes (*PA2G4, RPL24, RPS11, RPS16, RPS9*) and two subnetworks (scores >6) were discovered (Figure 9). where the genes in MCODE 1 were primarily involved in the ribosomal function-related gene cluster (Supplementary Figure 9A), *RSRC1, RPS(9, 17, 16, 15A, 11), RPL(36, 27, 24, 22, 15), MRPL(54, 49, 42, 37, 17)* and the gene clusters *PSM(D6, D13, C1, B9, B8, B6, PSMB3, B2)* that were involved in ubiquitinated degradation of proteins. PPI analysis of muscle-specific top 300 and fat-specific top 300 (Supplementary Figure S10) showed results in line with the enrichment analysis, with one MCODE (score = 6.2) found in a network containing a cluster of genes associated with actinomycosis: *MYH (2, 6, 7), MYL (2, 3, PF), ACT (A1, C1, N3), ATP2A1, CSRP3, SRL, TMEM38A, TNNC2*.

To sum up, integrating the enrichment and PPI analyses, it is clear that the common biological processes shared by MF regulatory axis were still dominated by energy metabolism and inflammation-related biological processes. However, the signaling pathways associated with inflammation in muscle and lipolysis in fat appear to be more active. Meanwhile, muscle is a predominantly energy-consuming motor tissue, and thereby genes and signaling pathways are closely associated with actin/myosin/tropomyosin.

## Discussion

Energy allocation and usage in animals is a systemic regulatory process that is operated in a coordinated multi-organ effort, which may be signaled to the associated regulatory axis to accomplish this complicated physiological process. The FE of an animal is determined by how the body allocates nutrients; if maintenance needs are increased, FE is decreased. The organism's organs should function in collaboration with each other, especially those involved in the digestion-absorption-production-consumption-storage process, and should be controlled by a set of sophisticated regulatory axis. Studies have revealed that different organs could communicate *via* the regulatory axis and determine the direction of nutrient flow, e.g., the brain-gut axis regulates obesity and appetite in animals (30) and maintains gastrointestinal glucose homeostasis (31). A novel vago-vagal liver-brain-gut reflex arc controls the number of pTreg cells and maintains gut homeostasis (32). As indicated by published data, the present study is the first to address the mechanisms of nutrient regulation by the rumen-liver-muscle- fat regulatory axis from a transcriptomic perspective.

The reciprocal interaction between the gastrointestinal tract and the liver is established by the portal vein, which transports nutrients absorbed by the gastrointestinal tract directly to the liver, where they then travel to other tissues that require energy consumption or storage (muscle, fat, etc.); the connecting system is linked by the vascular, gastrointestinal mucosal system (48). The rumen is an essential digestive organ for ruminants and is the principal site of microbial digestion; it is also the site of fermentation of volatile fatty acids (VFA) by the rumen microbiota that supplies 70% of the ruminant's daily energy (49). The liver is the pivotal organ of systemic metabolism [e.g., lipid metabolism, carbohydrate metabolism, protein metabolism (50)] and plays an essential role in maintaining lipid homeostasis (51), as well as being the site of a more active immune response, with bile acids secreted to facilitate the absorption of nutrients from the intestine (52). As the most energy-consuming organ of the organism, muscle directly determines the efficiency of energy usage in terms of FE. Meanwhile, fat tissue functions as a storage site for energy when the organism's maintenance needs are fulfilled. For this reason, it is essential to interpret the direction of energy flow along the RLMF regulatory axis. In the present work, we discovered a great similarity between gene expression and biological pathways in the **RLMF** regulatory axis, i.e., active expression of energy metabolism and inflammation-related genes. However, immune and lipid metabolism-related genes were more actively expressed in the liver than rumen, and transcriptional regulation-related genes and pathways were more active in the rumen than the liver.

FE is a complex quantitative trait controlled by polygenes (53). As the energy-consuming organs, it's speculated that genes related to the regulation of energy metabolism through the **RLM** regulatory axis might be responsible for the variation in RFI. We have identified a set of genes involved in the electron respiratory chain, which are core subunit components of the mitochondrial complex I-V and are up-regulated in the high FE group in the **RLM** regulatory axis, including *ND (2,3,4,4L,5,6), NDUF (A13, A7, S6, B3, B6), COX (1,3), CYTB, UQCR11, ATP (6,8)*. Previous studies of the high FE animals in cattle rumen (27) and skeletal muscle (26), chicken skeletal muscle (54), and swine skeletal muscle and liver (55, 56) also showed that genes associated with the electron respiratory chain are upregulated. These investigations all point to our supposition that the regulation of energy metabolism *via* the **RLM** regulatory axis may be one of the principal causes of FE variation. The electron respiratory chain is the primary site of energy production in the organism and can generate substantial levels of ATP. Therefore, the overall efficiency of the electron respiratory chain may be a potential determinant of FE. It has also been revealed that mitochondrial respiration rates were high FE than in high RFI individuals (57) and that mtDNA copy numbers were significantly lower, which exhibited lower uncoupling of the electron transport chain and oxidative stress (58, 59). Moreover, in skeletal muscle and liver, the expression of genes related to TCA (tricarboxylic acid) cycle and oxidative phosphorylation was relatively high (57, 60), and ATP was synthesized more efficiently (54, 61, 62). Hence, high **FE** organisms might have a more optimal layout of the electron respiratory chain, with less mitochondrial uncoupling, stress response, and mitochondrial number, which improves energy efficiency (**FE**).

The mediators of energy metabolism are based on the electron respiratory chain, but its substrates, **NADH** and FADH2, are primarily derived from carbohydrate metabolism, in which gluconeogenesis and lipid metabolism are predominant. In the rumen, a large amount of VFA (which provides 70% of daily energy requirements) was produced by microbial catabolism, then transported *via* the portal vein to the liver and served as raw materials for the synthesis of sugars or proteins (49). Therefore, it's speculated that the metabolic rate and efficiency of the relevant substances, particularly sugar, lipid, and protein, in the **LMF** regulatory axis might also be an essential contributor to **FE** variability. Consistent with anticipation, a set of genes closely related to lipid metabolism were found to be upregulated in the **LM** regulatory axis and **LF** regulatory axis: APO (A1, A2, A4, B, C3), *ALB, FG (A, G), HP, HPX, LPL, AMBP, FABP1, AHSG*. Our findings are concordant with the results in high **FE** chicken skeletal muscle (54) and pig liver and skeletal muscle (upregulation of the trans-lipoprotein gene family) (18, 56). It was also revealed that lipid synthesis-related genes were down-regulated and fatty acid oxidation-related genes were up-regulated in the livers of high FE pigs and ducks (22, 61). In addition, differential genes in pig and cattle skeletal muscle were mainly enriched in energy metabolism and lipid metabolism-related signaling pathways (26, 63). However, some other studies found that SCL was downregulated in pig and cattle liver of LRFI (61, 64, 65). As such, the efficiency of carbohydrate metabolism on the RLMF regulatory axis might be another prime contributor to **FE** variation.

Furthermore, protein metabolism-related genes are actively expressed in the **RLMF** regulatory axis, such as the *RPL* gene family (*8,18A,18,15,13, P1*), the *RPS* gene family (*23,27A,3A,4X*), and the *PSM* gene family (*A1-A7, B6, C1, C3, D2-D4, D8 D9, E1*). Ubiquitination of proteins suppresses immune and inflammatory responses (66) and enhances transcriptional activity due to the requirement to produce appropriate resistance proteins. In the present study, the **RLMF** regulatory axis has varying degrees of immune and inflammatory responses and protein ubiquitination, particularly in the liver and fat. Therefore, it is hypothesized that genes and pathways related to protein synthesis and catabolism are also effective in the regulatory axis involved. In the rumen of high **FE** cattle, the genes for protein synthesis (*RPL10, RPS15, RPL36*) and degradation (*PSMB6, UBC, UBA52, UBE2V1*) were significantly upregulated (27). In high **FE** pig's skeletal muscle, liver, dorsal , and perirenal adipose, the gene families *MRPL, MRPS, PRS, RPL, PSM* are upregulated (56), and protein ubiquitination was an active signaling pathway in the liver (28, 65). The upregulated genes in low **FE** chickens breast muscle were mainly involved in the immune and inflammatory responses (54). The highest differential expression enrichment in the pig liver were those involved in protein ubiquitination, followed by immunomodulation-related pathways (65). In the Angus cattle skeletal muscle transcriptome, differential genes were significantly enriched in immune response, inflammatory response, and muscle contraction/development (26). The immune and inflammatory response is highly energy-intensive, resulting in fewer nutrients available for its production (67). At the same time, it has been suggested that animals with high **FE** have a more efficient ability to resist inflammation and devote more energy to growth and muscle deposition (68). Therefore, the regulation of biological processes related to protein metabolism through the **RLMF** regulatory axis may contribute to the regulation of FE in animals.

To summarize, energy metabolism, substance metabolism, protein ubiquitination, inflammation, and immune-related signaling pathways on the **RLMF** axis are likely biological processes closely associated with **FE**, and genes enriched in these pathways might be involved in **FE** variation. Although we have integrated analysis of transcriptomic data from multiple tissues from a regulatory axis perspective to elucidate the potential contributors to **FE** variation, shortcomings also remain. First, the central nervous system is the regulatory center for feeding, and the arcuate nucleus (ARCs) of the hypothalamus is essential nuclei for sensing energy signals. We have not yet found transcriptomic data for brain, gut, liver, and fat in the same individual, which prevents us from confirming whether the hypothalamus also contains similar results. Next, we have only hypothesized that this regulatory axis is likely to exist based on the available studies, but functional experiments are lacking to establish a direct regulatory role. Meanwhile, more samples or similar studies are needed in other cattle breeds or species to confirm our results.

## Conclusion

The substance metabolism, energy metabolism, inflammation, and immune-related signaling pathways on the **RLMF** regulatory axis and the principal effector genes they contain may be the significant contributors to **FE** variation based on current data. Therefore, it is essential to incorporate a holistic approach (regulatory axis) into the breeding agenda for future molecular breeding activities in cattle.

## Data availability statement

The original contributions presented in the study are included in the article/Supplementary material, further inquiries can be directed to the corresponding authors.

## Author contributions

Conceptualization, supervision, and writing—review and editing: XK and YS. Software, visualization, and writing—original draft preparation: CY. Formal analysis: YD. Investigation: XD. All authors contributed to the article and approved the submitted version.
