# Supplementary figures and images for: Multi-transcriptomics reveals RLMF axis-mediated signaling molecules associated with bovine feed efficiency
Source: Front Vet Sci. 2023 Mar 22;10:1090517. doi: 10.3389/fvets.2023.1090517 (PMC10073569; doi:10.3389/fvets.2023.1090517)

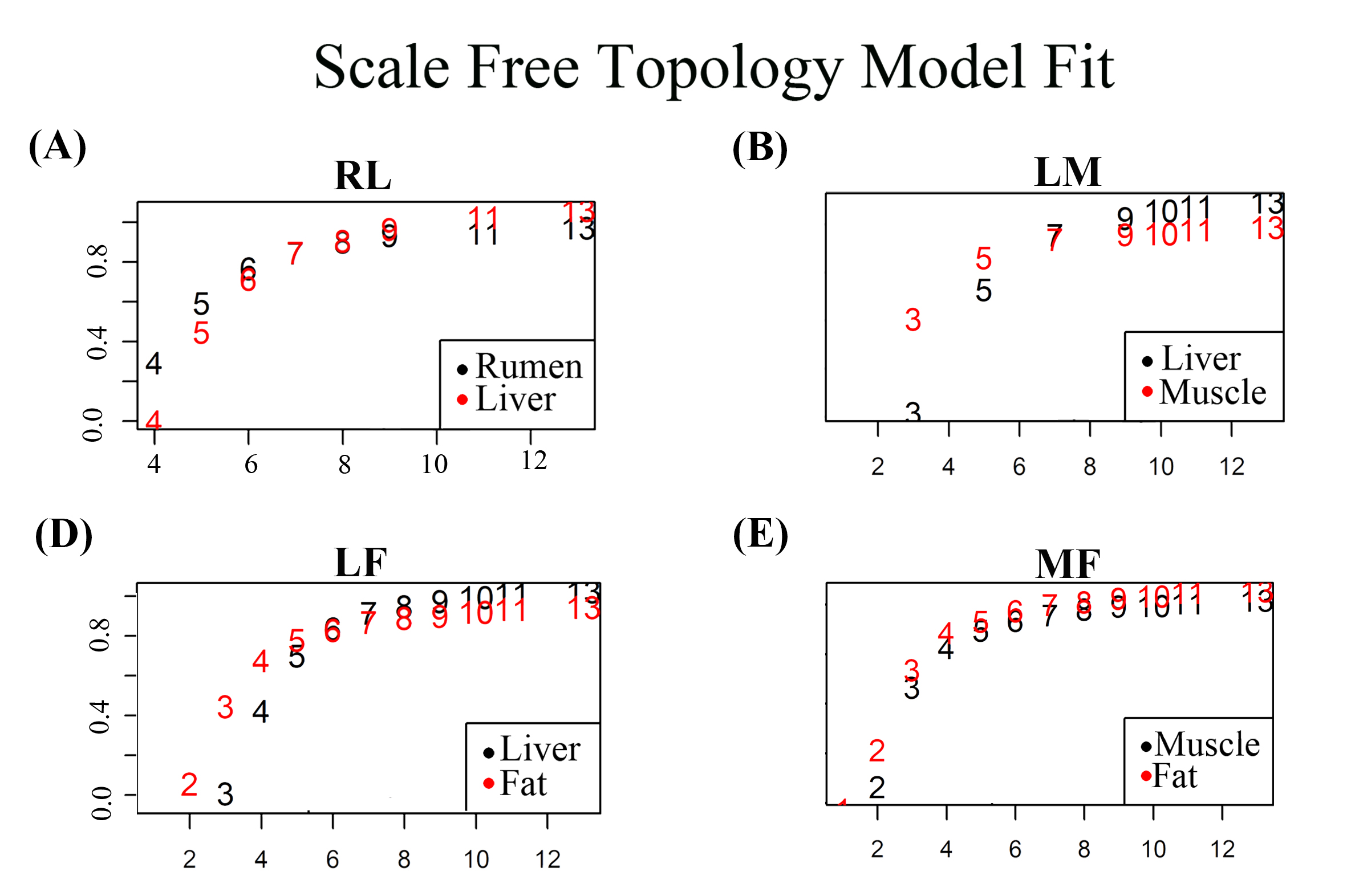

Supplement: Supplementary file 3 [file Image_1.JPEG]

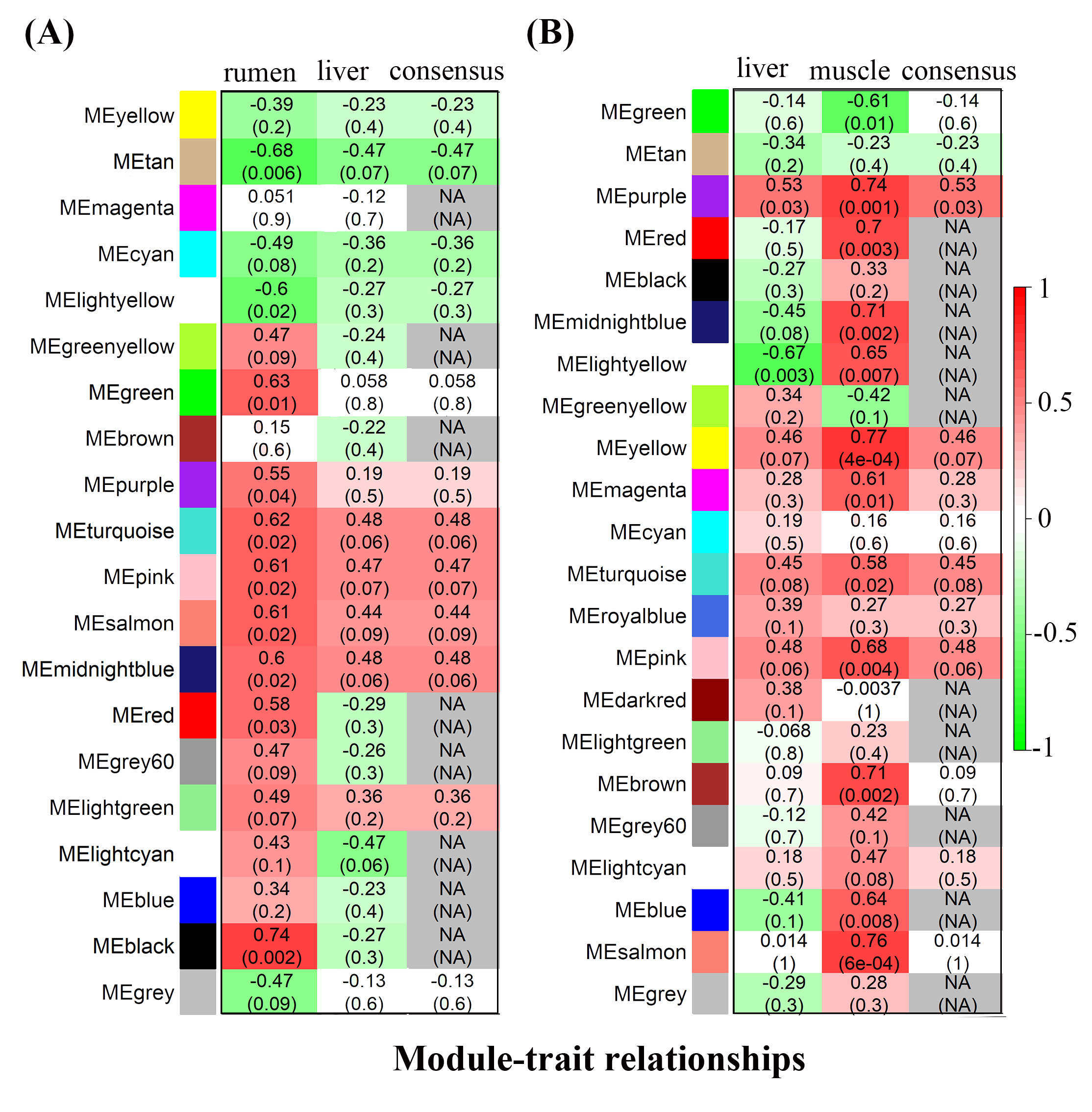

Supplement: Supplementary file 4 [file Image_2.JPEG]

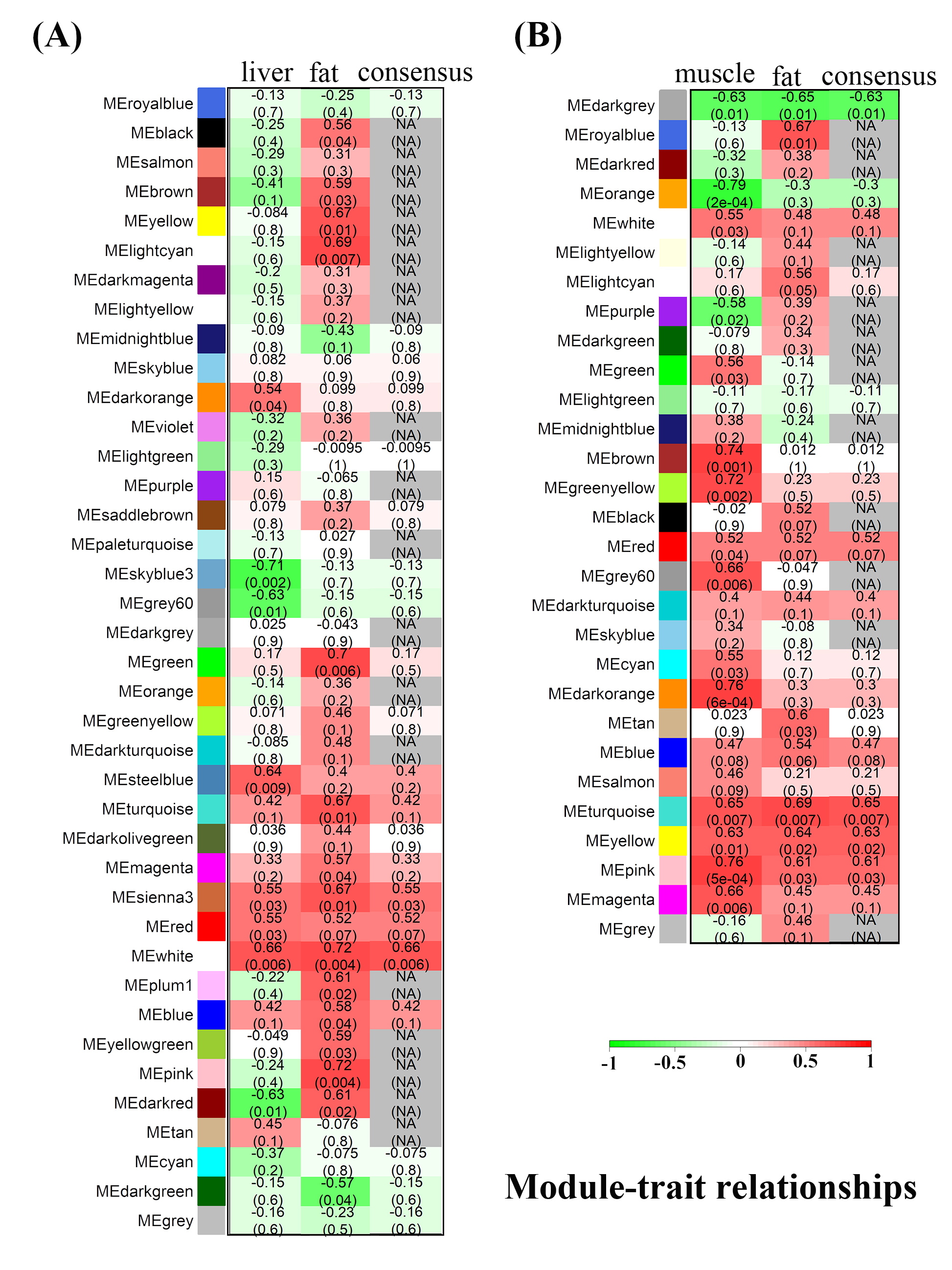

Supplement: Supplementary file 5 [file Image_3.JPEG]

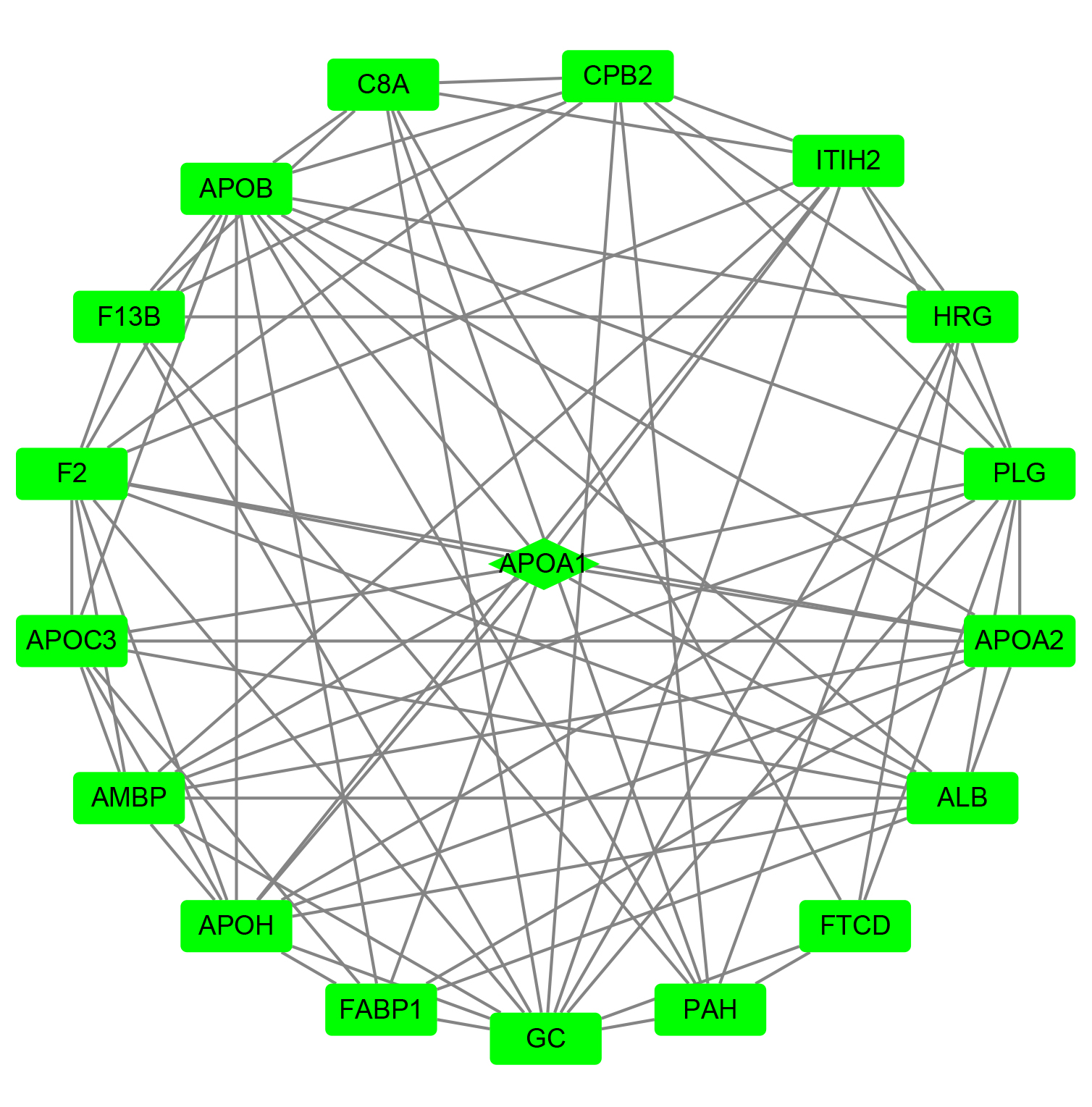

Supplement: Supplementary file 7 [file Image_5.JPEG]

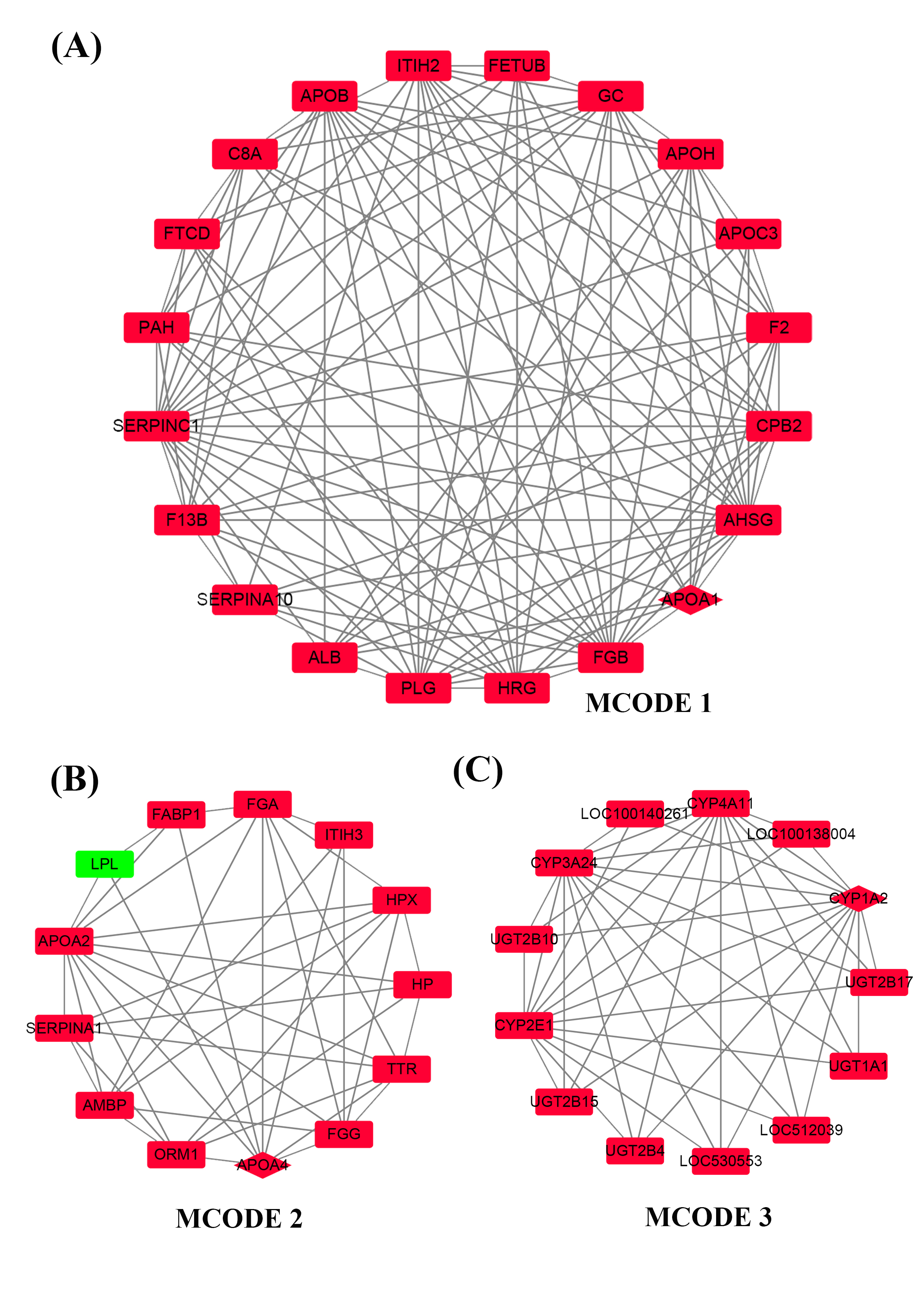

Supplement: Supplementary file 9 [file Image_7.JPEG]

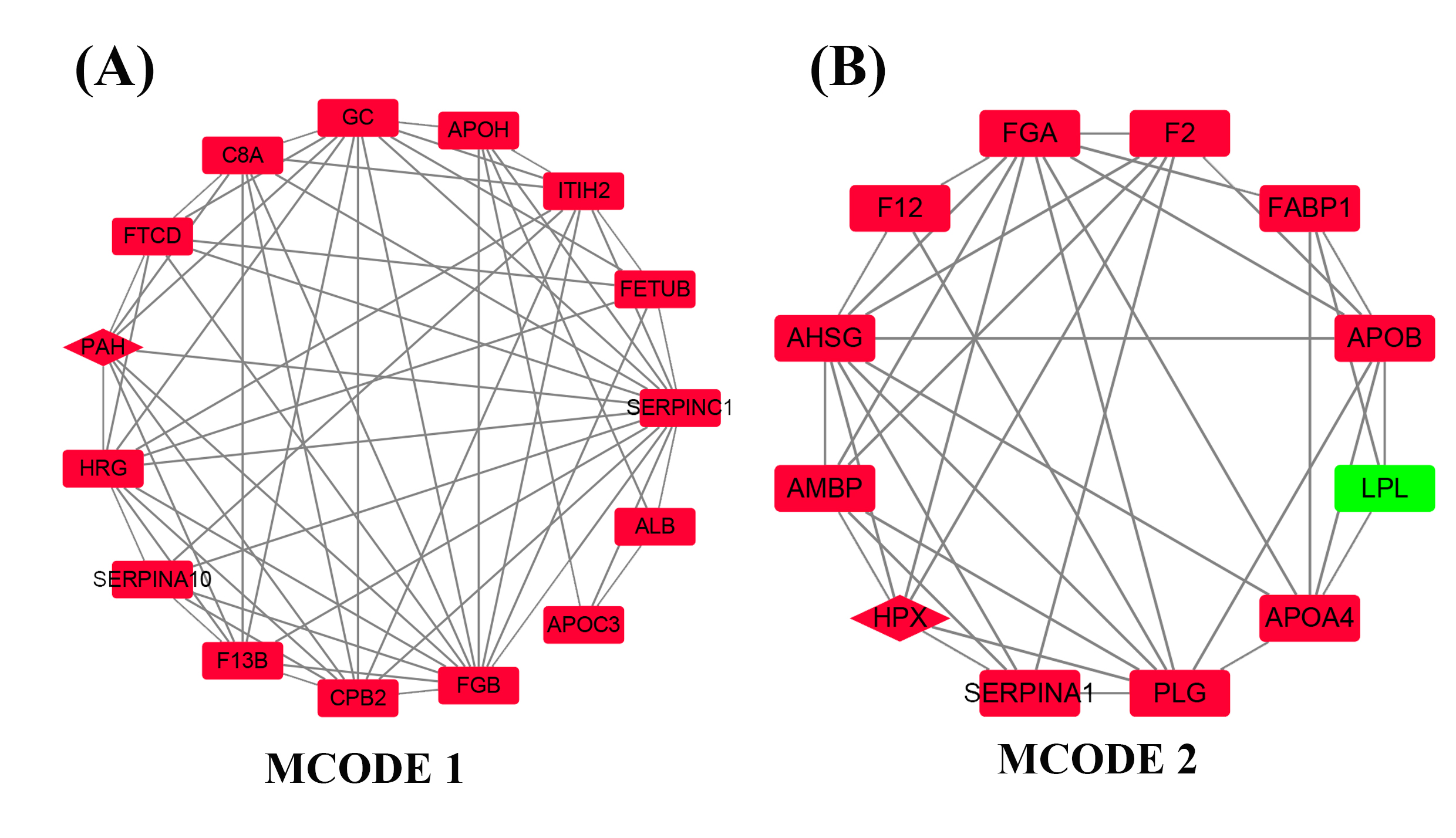

Supplement: Supplementary file 11 [file Image_9.JPEG]
